# Care for MRSA carriers in the outpatient sector: a survey among MRSA carriers and physicians in two regions in Germany

**DOI:** 10.1186/s12879-016-1503-5

**Published:** 2016-04-26

**Authors:** Heike Raupach-Rosin, Nicole Rübsamen, Sebastian Szkopek, Oliver Schmalz, André Karch, Rafael Mikolajczyk, Stefanie Castell

**Affiliations:** Department Epidemiology, Helmholtz Centre for Infection Research, Inhoffenstr. 7, 38124 Braunschweig, Germany; PhD Programme “Epidemiology”, Braunschweig-Hannover, Germany; Städtisches Klinikum Braunschweig, Institut für Mikrobiologie, Immunologie und Krankenhaushygiene, Braunschweig, Germany; Helios Klinikum Wuppertal, Abteilung für Onkologie und Palliativmedizin, Heusnerstraße 40, 42283 Wuppertal, Germany; German Centre for Infection Research, Hannover-Braunschweig, Germany; Hannover Medical School, Hannover, Germany

**Keywords:** MRSA, Stigmatization, Outpatient sector, Decolonization

## Abstract

**Background:**

Little is known about the management of methicillin-resistant *Staphylococcus aureus* (MRSA) carriers in the German outpatient sector and about the impact of MRSA on their daily life. Reimbursement for MRSA related costs in the German outpatient sector is available since 2012, but its impact has not been studied yet. The aim of the study was to analyze the outpatient management of MRSA carriers from both, physicians’ and MRSA carriers’ perspective.

**Methods:**

Paper-based questionnaires were mailed to physicians providing outpatient care and to MRSA carriers in 2013. MRSA carriers were recruited among patients tested positive for MRSA during a hospital stay in 2012. General practitioners, specialists for internal medicine, urologists, and dermatologists working in the outpatient catchment areas of the hospitals were contacted.

**Results:**

Out of 910 MRSA carriers 16.5 % completed the questionnaires; among 851 physicians 9.5 % participated. 27.3 % of the responding MRSA carriers stated that no healthcare professional had ever talked to them about MRSA. 17.4 % reported self-stigmatization in terms of restricting social contacts; 47.3 % remembered decolonization and 33.3 % reported that their MRSA status was checked after discharge. Physicians displayed heterogeneous attitude and activity towards MRSA (number of applied decolonization and MRSA screenings). A minority (15.2 %) were satisfied with the reimbursement of costs, 35.9 % reported full agreement with the general recommendations for the handling of MRSA carriers.

**Conclusions:**

MRSA carriers appear not well informed; (self-) stigmatization is occurring and should be tackled. Greater awareness of MRSA as a problem in the outpatient sector could lead to a better handling of MRSA carriers.

**Electronic supplementary material:**

The online version of this article (doi:10.1186/s12879-016-1503-5) contains supplementary material, which is available to authorized users.

## Background

Methicillin-resistant *Staphylococcus aureus* (MRSA) has a prevalence of around 2.2 % in newly admitted patients to German hospitals [1] and can be detected in 18–20 % of inpatients’ derived *Staphylococcus aureus* isolates [2]. To diminish the overall load of MRSA, a bundle of measurements is recommended both, on the hospital level (e.g., isolation) and on the individual level (e.g., decolonization therapy) [3]. While the spread of MRSA is especially problematic in the inpatient sector, transmissions can be initiated by MRSA carriers readmitted to the hospital. In order to interrupt the MRSA transmission, not only measures in the hospital but also follow-up and decolonization of patients in the outpatient sector is necessary. Meyer et al. [4] reported that decolonization therapy can be applied successfully in the outpatient sector and pointed out that a close cooperation between outpatient and inpatient sector is necessary. However, a follow-up system for MRSA carriers (or carriers of other multiresistant pathogens) across sectoral borders (inpatient vs. outpatient) is missing in Germany, and we could not find any international studies concerning this subject. The German College of General Practitioners and Family Physicians (DEGAM) had published guidelines for the diagnosis and therapy of MRSA in the outpatient sector in September 2013 (three months before the survey). These recommendations are in accordance with the ones of the KBV (National Association of Statutory Health Insurance Physicians) published earlier. Both recommend MRSA screening for patients with an increased risk of being MRSA-positive, as well as three control swabs 48 h, 3–6 months and 12 months after application of a decolonization therapy. This treatment is recommended for all MRSA carriers; however, in case of factors that might decrease the success such as chronic wounds, the physician can opt out. The guidelines recommend up to two decolonization therapies before consulting a specialist, e.g., a MRSA network. The treatment should comprise a bundle of measures (nasal ointment, mouthwashes, and daily disinfection of hair and skin for 5 days). Accompanying measures should include daily change of clothes, bedding and towels, and disinfection or daily change of hygiene utensils [5].

In order to improve treatment of MRSA in the outpatient sector, reimbursement of the costs for MRSA screening, control swabs, and decolonization therapy was introduced in April 2012 by the Federal Joint Committee (“Gemeinsamer Bundesausschuß”), and the reimbursement is now paid by statutory health insurances. It is noteworthy, that only the costs for nasal ointment (mupirocin) are covered by the statutory health insurances. The impact of this reimbursement has not been studied yet.

In addition, risk perceptions regarding MRSA are likely to cause stigmatization of MRSA carriers, as was described for the UK [6] and for Sweden [7]. Up to now, nothing is known about the perception of stigmatization due to MRSA in the daily life of MRSA carriers after discharge from hospital in Germany [8]. Greiner [9] demonstrated a considerable loss of quality of life for patients with MRSA infection, but no data are available about the quality of life of MRSA carriers without symptoms of MRSA infection.

Therefore, our study aimed at assessing knowledge, attitude, and practice related to MRSA among MRSA carriers and among physicians in outpatient care in two regions of Germany after the introduction of an additional reimbursement for MRSA specific care. In addition, we studied the perceived stigmatization and quality of life in MRSA carriers. Beside this quantitative approach, we initiated focus groups with MRSA carriers from the same study population [10].

## Methods

### Study population

MRSA carriers were recruited in collaboration with two tertiary care hospitals in Lower Saxony and North Rhine-Westphalia in November and December 2013. Inclusion criterion was a positive MRSA test during a hospital stay in 2012, that is 12 to 24 months before initiation of the study. For simplification, we call these patients “MRSA carriers”, regardless of their current MRSA status and if they initially had a MRSA colonization or a MRSA infection. The questionnaires were mailed to their home addresses from the hospitals. The questionnaire for physicians was sent to all general practitioners (GPs), specialists for internal medicine, dermatologists, and urologists in the catchment area of the two hospitals between October and November 2013. We focused on these specialities in order to query physicians who presumably frequently deal with MRSA positive patients.

### Ethics Statement

All questionnaires were filled in anonymously by the participants. The study was approved by the Ethics committees of Hannover Medical School (No. 1893–2013) and the University Witten/Herdecke (No. 112/2013).

### Questionnaires

#### Physicians in outpatient care

We developed a questionnaire assessing relevance of MRSA in their practices (11-point scale), knowledge of the refunding possibilities for MRSA screening and treatment, and satisfaction with financial reimbursement for MRSA specific care. Knowledge of MRSA was evaluated by a cumulative score; for further analysis, we dichotomized this knowledge score (lower group 0–3 points, higher group 4–7 points). To assess their activity regarding MRSA, we asked how many screening tests and decolonization therapies they applied and if they are members of a MRSA network which is a regional quality management measure for training and discussions on MRSA. The questionnaire was piloted for clarity and comprehension with three GPs and one specialist in internal medicine working in the outpatient sector. The translated version of the questionnaire for physicians is available as Additional file 1.

#### MRSA carriers

Analogously, we developed a questionnaire for MRSA carriers, including questions on MRSA history, general state of health, perceived stigmatization, and socio-demographic data. As among physicians, knowledge of MRSA was assessed by a cumulative score based on 7 items and dichotomized for further analysis (lower group 0–3 points, higher group 4–7 points, one point for every correct answer). Two questions focused on the patients’ attitudes towards MRSA, namely the importance respondents attributed personally to MRSA, and if they were scared of MRSA, with answer categories on a 5-point Likert scale. We reclassified the responses into two categories (high:“yes a lot” and “yes, some” vs. low:“neutral”, “rather not” and “not”). Furthermore, we included questions on decolonization and control swabs as well as eight questions about perceived constraints in daily life and perceived stigmatization. The translated version of the questionnaire for MRSA carriers is available as Additional file 2. We added one question on the self-rated health status which was previously used as a single item in several studies, e.g., in the 1998 German Federal Health Survey [11]. The questionnaire was piloted with four healthy adults for comprehension.

#### Statistical analysis

Differences between groups were tested using the chi-squared test for categorical variables and the Wilcoxon rank sum test for continuous variables; additionally the odds ratio with 95 % confidence intervals is indicated for the univariable analysis. For explorative multivariable analysis of MRSA related knowledge, we used the dichotomized knowledge score as outcome variable for both, MRSA carriers and physicians and applied logistic regression. Variables with *p* < 0.25 in the univariable analysis as well as age and sex were included in an automatic forward selection model building procedure (using as cutoff *p* = 0.2 for inclusion of variables and *p* = 0.05 for exclusion, based on the Wald-Test). The Hosmer–Lemeshow test was used to test the goodness of fit of the logistic models. The analysis was carried out using Stata 12 (StataCorp, College Town, US).

## Results

### Physicians in the outpatient sector

The response proportion to our questionnaire for physicians was 9.5 % (80/851), 27.8 % of them being female and 38.0 % being GPs (Table 1).

**Table 1:** Characteristics of responding physicians

	*N* (%^a^)
Total	79 (100 %)
Age, *n* = 75	
Median (IQR) in years	52 (46–58)
Sex, *n* = 74	
Female	22 (27.8 %)
Years of professional experience in ambulant health care	
Median (IQR) in years, *n* = 74	13.5 (3–34)
Estimated number of MRSA positive patients in the last 12 months	
Median (IQR), *n* = 77	5 (3–10)
Number of screened patients in the last 12 months	
Median (IQR), *n* = 78	2 (0–6)
Number of decolonized patients ever	
Median (IQR), *n* = 79	2 (0–7)
Discipline, *n* = 75	
General practitioner	30 (38.0 %)
Internal medicine	30 (38.0 %)
Dermatologist	4 (5.1 %)
Urologist	8 (10.1 %)
Other	3 (3.8 %)

*IQR* interquartile range

^a^ Calculation of proportions includes missing values in the denominator

Only 57.0 % of the physicians were able to correctly define a patient being at risk for MRSA according to the definition issued by the ‘National Association of Statutory Health Insurance Physicians’, 51.9 % knew at which time points control swabs are recommended and 14.0 % answered all questions about the reimbursement correctly. Respondents achieved a median of four knowledge points out of seven possible. In the multivariable analysis regarding factors associated with knowledge related to MRSA, only the variable "relevance" was selected in the model, physicians attributing higher relevance to MRSA answered more questions correctly (odds ratio (OR) 1.4 per one point increase, 95 % confidence interval (95 % CI) 1.1 to 1.7, *p* = 0.002) (Table 2). Those displaying more activity towards MRSA showed better knowledge in the univariable analysis. However, this was not significant in the multivariable model.

**Table 2:** Variables associated with physicians’ knowledge related to MRSA

	Univariable analysis				Multivariable analysis^b^	
	Less knowledge (0–3 points)	More knowledge (4–7 points)	*p*	OR (95 % Confidence interval)	OR (95 % Confidence interval)	*p* (Wald test)
	*n* (%)^a^	*n* (%)^a^				
	36 (45.6 %)	43 (54.4 %)				
Age			0.701	0.8 (0.5–1.4) per ten years increase		
Median (IQR)	52 (46–59)	52 (45–57)				
Sex			0.090			
Female	13 (39.4 %)	9 (21.4 %)		0.4 (0.1–1.1)		
Male	20 (60.6 %)	33 (78.6 %)		1		
Professional experience in years			0.909	0.9 (0.6–1.5) per 10 years increase		
Median (IQR)	13 (8–24)	14.5 (8–22)				
Discipline			0.163			
General practitioner	24 (72.7 %)	24 (57.1 %)		1		
Other specialist	9 (27.3 %)	18 (42.9 %)		2 (0.7–5.3)		
Member of a MRSA-Network			0.135			
Yes	2 (5.6 %)	7 (16.3 %)		3.3 (0.6–17.0)		
No	34 (94.4 %)	36 (83.7 %)		1		
MRSA–certificate			0.005			
Yes	8 (22.2 %)	23 (53.5 %)		4.0 (1.5–10.8)		
No	28 (77.8 %)	20 (46.5 %)		1		
Subjectiv relevance for physician’s work			0.001	1.3 (1.1–1.6) per one point increase	1.4 (1.1–1.7) per one point increase	0.002
Median (IQR)	3.5 (1.5;7)	7 (5;8)				
Number of MRSA carriers last 12 months			0.030	1.7 (0.9–3.3) per increase of 10		
Median (IQR)	5 (1.5;7)	6 (4;10)				
Number of screenings in the last 12 months			0.019	1.1 (0.8–1.6) per increase of 10		
Median (IQR)	2 (0;4)	3 (1;10)				
Number of decolonizations			0.0625	1.4 (0.8–2.5) per 10 increase		
Median (IQR)	2 (0;4.5)	4 (1;10)				
Satisfaction with refunding			0.008			
Content	1 (3.9 %)	11 (26.2 %)		1		
Discontent	7 (26.9 %)	17 (40.5 %)		0.2 (0.0–2.0)		
Don’t know	18 (69.2 %)	14 (33.3 %)		0.1 (0.0–0.6)		

^a^ Differences to total *N* due to missing values

^b^ Logistic regression with forward selection of variables; mutually adjusted for all variables with reported ORs in the table

Nearly half of the physicians (45.6 %) stated that “Sufficient information about MRSA is available”. One third (35.9 %) agreed fully with the general recommendations about handling of MRSA carriers and another 46.2 % agreed with them “in part”.

According to our respondents, MRSA findings are not always reported in the discharge documents: 59.0 % answered the findings were “often” or “very often” reported, 28.2 % “sometimes”, and 12.8 % answered “seldom” or “never”. A notification of a MRSA finding by telephone was even less common: 5.2 % answered “often”, 10.4 % “sometimes”, 84.4 % “seldom” or “never”. Importance attributed to MRSA by the physicians was fairly heterogeneous: 22.1 % attributted low importance (0–2 points/11), and 32.5 % attributed high importance (9–10/11).

Physicians reported that they screened 2 patients (median) for MRSA in the last 12 months. Furthermore, they have initiated a median of 2 decolonization therapies ever (Table 1); nearly one third of them (27.9 %) has never applied a decolonization therapy to a patient while 22.8 % have applied it 10 times or more. Fifty–eight percent of the responding physicians were aware of the refunding possibilities and satisfaction with the amount of refunding was distributed as follows: no participant was “very satisfied”, 15.2 % were “satisfied”, 30.4 % were “not satisfied”, 54.4 % answered “I don’t know”or did not answer this question.

### MRSA carriers

Based on hospital records, 2250 MRSA carriers were eligible for our study. Of these, we excluded 1089 because their death was known or assumed by the hospital, and 251 letters were undeliverable. Of the remaining 910 MRSA carriers, 16.5 % (150) sent back a completed questionnaire (Additional file 3: Figure S1). MRSA carriers appeared as a frail study population with a median age of 71.5 years; 33.3 % reported to have been assigned a formal long-term care level for purposes of German health insurance, which corresponds to a considerable and long-term need of nursing care (Table 3).

**Table 3:** Characteristics of MRSA carriers

	*N* (%^e^)
Total	150 (100 %)
Age, *n* = 146	
Median (IQR) in years	71.5 (60–78)
Sex, *n* = 146	
Female	67 (44.7 %)
Education, *n* = 136	
Low^a^	83 (55.3 %)
Intermediate^b^	30 (20.0 %)
High^c^	23 (15.3 %)
Living in a long term care facility, *n* = 141	
Yes	9 (6.0 %)
No	132 (88.0 %)
Need of nursing care (“Pflegestufe”), *n* = 144	
Yes	50 (33.3 %)
No	94 (62.7 %)
Migration Background^d^, *n* = 147	
Yes	15 (10.0 %)
No	132 (88.0 %)
Risk factors for MRSA (multiple selection possible), *n* = 150	
Urinary catheter	9 (6.0 %)
Dialysis	10 (6.7)
Chronic wounds	13 (8.7 %)
Chronic skin disease	12 (8.0 %)
Occupational exposure to lifestock	1 (0.7 %)
No risk factor	116 (77.3 %)
Decontamination therapy applied, *n* = 132	
Yes	71 (47.3 %)
in the hospital	44 (29.3 %)
at home	21 (14.0 %)
in the hospital and at home	29 (19.3 %)
No	61 (40.6 %)
Control swabs for MRSA in the outpatient sector, *n* = 144	
Yes	50 (33.3 %)
Control swab and decolonization therapy was applied, *n* = 132	
Yes	37 (24.7 %)

^a^Low level of school education (<10 years)

^b^Intermediate level of school education (10–12 years)

^c^High level of school education (12–13 years)

^d^Migration defined as not being born in Germany or/and mother tongue not German

^e^ Calculation of proportions includes missing values in the denominator

*IQR* interquartile range

**Fig. 1:**
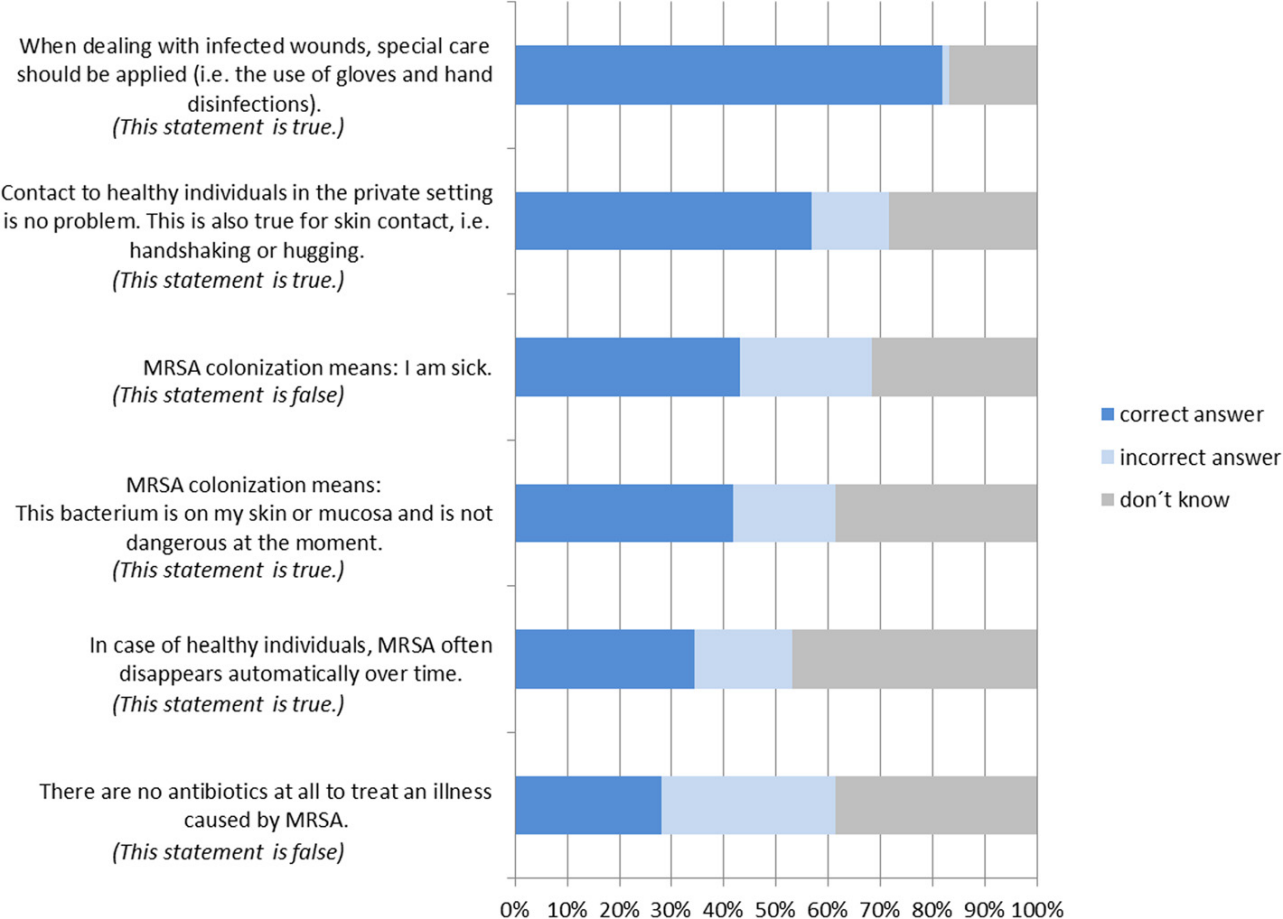
Knowledge of MRSA carriers

To seven knowledge questions, MRSA carriers gave a median of three correct answers; a majority (64.0 %) answered “don’t know” to at least one question (Fig. 1). In the multivariable analysis, those participants who had sought additional information through internet (OR 5.1; 95 % CI 1.8 to 14.0, *p* = 0.002) or newspapers (OR 5.4; 95 % CI 1.6 to 17.5, *p* = 0.005) showed significantly more knowledge about MRSA. Older MRSA carriers had less knowledge (OR 0.7 per 10 year increase, 95 % CI 0.5 to 1.0, *p* = 0.049) as well as those participants attaching more importance to MRSA (OR 0.4, 95 % CI 0.2 to 0.9, *p* = 0.034). Interestingly, education level was not associated with knowledge related to MRSA (Table 4).

**Table 4:** Variables associated with MRSA carriers’ knowledge related to MRSA

	Univariable Analysis				Multivariable Analysis^b^	
	Less knowledge (0–3 points)	More knowledge (4–7 points)	*p*	OR (95 % confidence interval)	OR (95 % confidence interval)	*p*
	*n* (%)^a^	*n* (%)^a^				
	82 (54.7 %)	68 (45.3)				
Age				0.7 (0.5–0.9) per 10 year increase	0.7 (0.5–1.0) per 10 year increase	0.049
Median (IQR) in years	74 (65–79.5)	66 (53–77)	0.011			
Sex						
Female	35 (43.8 %)	32 (48.5 %)	0.568	1.2 (0.6–2.3)		
Male	45 (56.3 %)	34 (51.5 %)		1		
Education						
Low	53 (72.6 %)	30 (47.6 %)	0.011	1		
Intermediate	12 (16.4 %)	18 (28.6 %)		2.6 (1.1–6.2)		
High	8 (11.0 %)	15 (23.8 %)		3.3 (1.3–8.7)		
Migration Background						
Yes	11 (13.9 %)	4 (5.9 %)	0.108	0.4 (0.1–1.3)		
No	68 (86.1 %)	64 (94.1 %)		1		
Source of Information						
Internet						
Yes	9 (11.0 %)	25 (36.8 %)	<0.0001	5.4 (2.0–11.0)	5.0 (1.8–14.0)	0.002
No	73 (89.0 %)	43 (63.2 %)		1	1	
Newspaper						
Yes	8 (9.8 %)	14 (20.6 %)	0.015	2.4 (0.9–6.1)	5.4 (1.6–17.5)	0.005
No	74 (90.2 %)	54 (79.4 %)		1	1	
Television/Radio						
Yes	7 (8.5 %)	13 (19.1 %)	0.058	2.5 (0.9–6.8)		
No	75 (91.5 %)	55 (80.9 %)		1		
MRSA discussed with healthcare professional						
At least one	54 (65.9 %)	55 (80.9 %)	0.040	2.2 (1.0–4.7)		
None at all	28 (34.2)	13 (19.1 %)		1		
Attitude: Importance of MRSA						
High	51 (68.0 %)	35 (51.5 %)	0.004	0.5 (0.3–1.0)	0.4 (0.2–0.9)	0.034
Low	24 (32.0 %)	33 (48.5 %)		1	1	
Attitude: Scared of MRSA						
Scared of MRSA	46 (59.0 %)	29 (42.7 %)	0.049	0.5 (0.3–1.0)		
Not scared of MRSA	32 (41.0 %)	39 (57.4 %)		1		

^a^ Differences to total N due to missing values

^b^ Logistic regression with forward selection of variables; mutually adjusted for all variables with reported ORs in the table

About a quarter of the respondents (27.3 %) stated that no professional healthcare worker has talked to them about MRSA. The remaining reported more often talking with hospital staff (physicians or nursing staff) than with physicians or nurses in the outpatient care service (data not shown). Twenty-one percent reported to attribute no importance to the positive MRSA result, but 51.0 % were scared of MRSA.

Half of the respondents (49.3 %) reported that their general state of health was “not so good” or “bad”, and 20.0 % of the participants reported a deterioration of their quality of life due to MRSA.

**Fig. 2:**
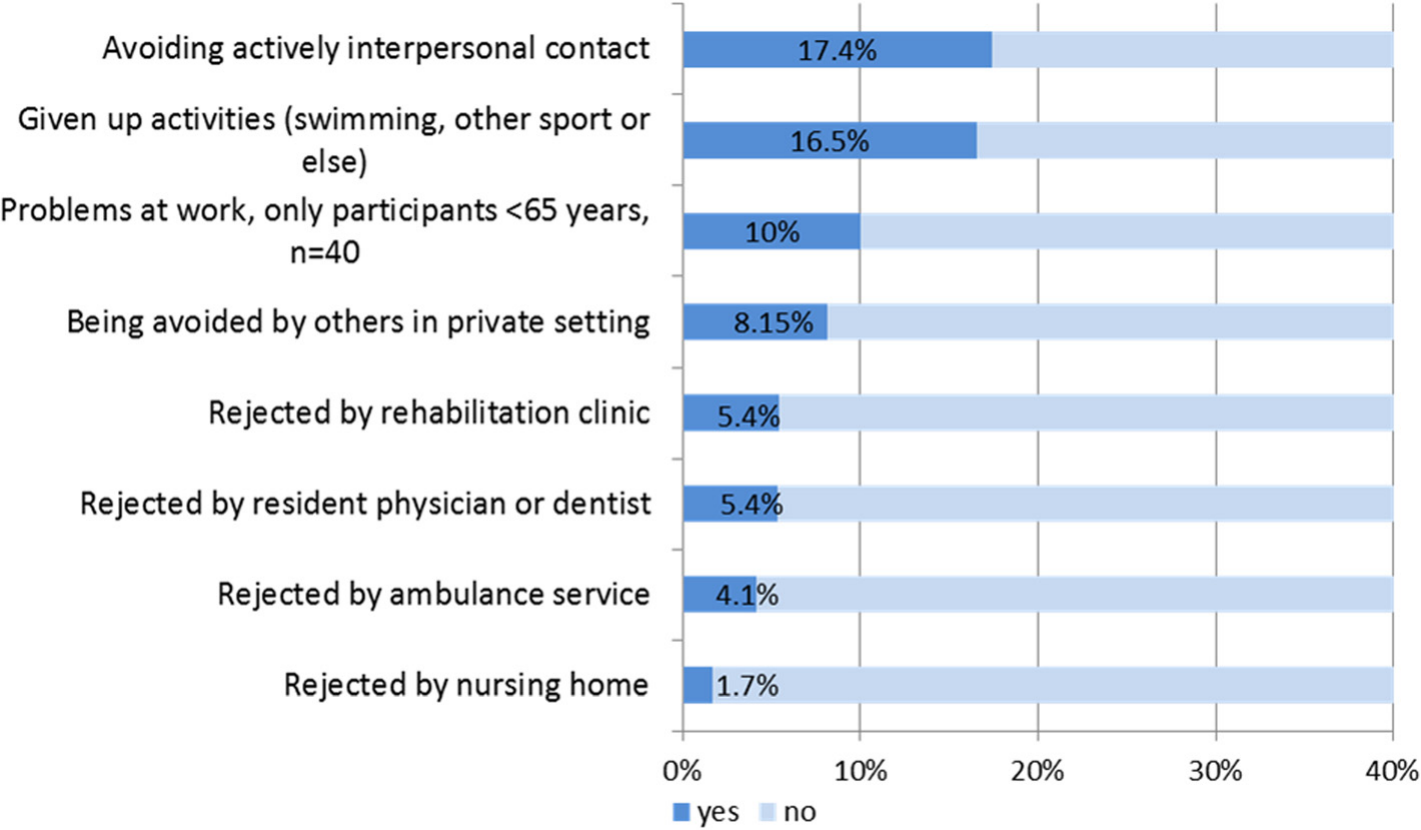
Stigmatization related to MRSA

One third (30.7 %) responded affirmatively to at least one question indicating stigmatization, and the aspect the most frequently reported was self-restriction of social contacts in order to prevent transmission (17.4 %). Of patients younger than 65 years (which was retirement age in Germany), 10 % (4/40) reported occupational problems because of MRSA. Participants also reported stigmatization in the context of health care services (Fig. 2).

Only one third (33.3 %) reported that their MRSA status was evaluated after discharge from hospital. Nearly half of the respondents (47.3 %) received a decolonization therapy, and of those, 52.1 % (37/71) reported that a control swab was taken in the outpatient sector (Table 3).

**Fig. 3:**
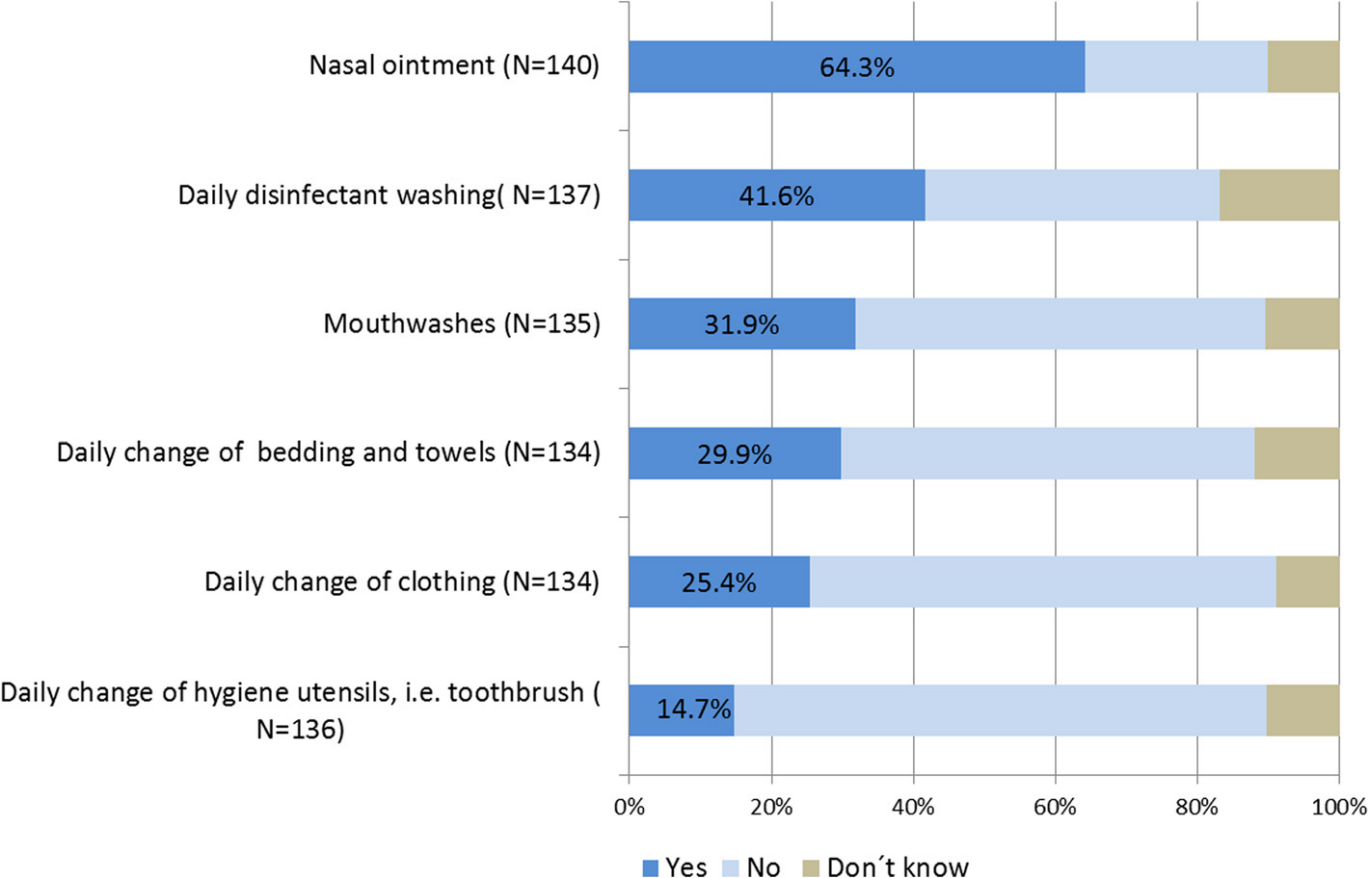
Decolonization therapy: Application of single measures

Asked about the application of the recommended measures in detail, only ten participants (6.7 %) stated that all listed measures had been applied; the application of nasal ointment being the most common (64.3 % of all MRSA carriers) (Fig. 3). According to our data, the presence of self-reported risk factors for prolonged MRSA carriage like chronic wounds or urinary catheters did not influence the application of decolonization therapy (Chi^2^ test, *p* = 0.636).

## Discussion

We analyzed knowledge, attitude and practice among MRSA carriers and physicians in the outpatient sector in Germany after the introduction of reimbursement for MRSA related therapy. Physicians displayed heterogeneous knowledge and level of activity regarding MRSA specific aspects. Almost one third of the responding MRSA carriers stated that no healthcare professional had ever talked to them about MRSA. Thirty percent claimed that their quality of life deteriorated due to MRSA and one third experienced stigmatization due to MRSA or exerted self-stigmatization.

Similarly,the reduction of social contacts and leisure activities among MRSA carriers was also reported in a Swedish study [7] and in our own focus groups, where participants reported the reduction of social contacts, e.g., to their grandchildren as a consequence of the MRSA finding [10]. Such behaviour is generally not recommended in official guidelines [12]. Nevertheless, some MRSA carriers seem to overestimate the risk associated with MRSA for healthy and non-hospitalized individuals. It might be advisable to proactively address this topic in patient information leaflets and physician-patient consultations.

Some participants reported a rejection by health care services like a nursing home or a rehabilitation clinic because of MRSA. Further studies are necessary to investigate to which extent the medical treatment of the underlying conditions is negatively influenced by a positive MRSA status.

MRSA carriers who sought additional information about MRSA on the internet or by reading newspapers could answer more knowledge questions correctly. This suggests that publicly available information on MRSA is used by MRSA carriers and has a positive effect on their knowledge. Hence, the quality and availability of public information on MRSA is important. Thirty percent of the respondents claimed that no healthcare professional had ever talked to them about MRSA. Astonishingly, some of these also reported the application of a decolonization therapy. In these cases, either the decolonization therapy has been applied without a proper clarification or the responding MRSA carriers did not remember the education about MRSA. Respondents gave, however, more inconsistent answers in this area: only 71 respondents reported at least one decolonization therapy, whereas 90 reported the application of nasal ointment. These contradictions also underline the lack of specific knowledge of the MRSA carriers. Around 30 % of the answers to the knowledge questions were “don’t know”, which indicates rather substantial deficits in the knowledge among MRSA carriers. The need for more information was also a key finding in qualitative studies from Great Britain and Sweden [6, 7]. Participants of our qualitative part of the study also reported a need for more and adequate information [10]. The regulation of the German National Association of Statutory Health Insurance allows reimbursement for ten minutes of conversation about MRSA twice during the treatment process [13]. Taking the complexity of the topic into account, it seems challenging to give adequate information in ten minutes, which could in part explain the information deficit of the MRSA carriers.

Rather surprisingly, 42 % of the responding physicians stated that there was not enough information available on MRSA. Various organizations in Germany (Robert Koch- Institute, Association of Health Insurance Doctors etc.) have detailed, freely available information on MRSA on their websites. In order to raise awareness of MRSA among physicians in the outpatient sector, it might not be sufficient to make information available through internet but it might be essential to additionally spread information proactively through medical journals or leaflets.

Only one third (33.3 %) of the MRSA carriers in our study reported to have received control swabs in the outpatient sector. This can be put in the context to the results of the survey among physicians, which showed a huge heterogeneity in their activity towards MRSA. Correspondingly, only half of the MRSA carriers reported a decolonization therapy, even though it was recommended for all patients except those with underlying conditions like, e.g., dialysis. However, the application of a decolonization therapy was not associated with the presence of these conditions among patients in our study population. So why did only half of the patients get a decolonization therapy? One reason could be that MRSA diagnosis is not always reported in the discharge letter. According to a recent report of the European Observatory on Health Systems and Policies “communication between GPs and hospitals is problematic” in Germany [14] as well as in some other European countries [15]. According to this report, in Spain, e.g., the cooperation between sectors is standard practice and has been facilitated by shared electronic clinical record and IT systems [14]. One possibility to overcome the sectoral gap might be the establishment of infection control teams taking care of the diagnosed MRSA carriers beyond the discharge from hospitals. Furthermore, it might be difficult for the physicians to develop routines with regard to MRSA due to the small number of cases. Kock et al. [2] estimated 132,000 MRSA cases in German hospitals per year; distributing them on the 37,353 general practitioners (GPs) in Germany in 2013 [16], one GP would see on average 3.5 MRSA carriers per year. This is quite comparable to the median of 5 MRSA carriers that were reported by the physicians in our data. This small number might result in a vicious circle: physicians attach little importance to MRSA, do not seek information about MRSA, and underestimate the number of patients at risk for MRSA, therefore apply screening measures too seldom, and consequently have low numbers of MRSA carriers among their patients.

Only few respondents reported that all recommeded measures (disinfectant washing etc.) were applied. One reason could be that only the costs for nasal ointment are covered by the health insurances, the carriers having to bear the rest of the costs themselves. The other reason could be, that physicians do not believe all the measures to be necessary: only one third (36 %) agreed fully with the general recommendations. The poor adherence and low agreement with the recommendations might be due to a lack of studies showing the effectiveness of the various recommended measures as was highlighted before by Faetkenheuer et al. [17]; however, we have no insight from our study to which aspects of the recommendations exactly the physicians do not agree and why. Studies exploring the effectiveness of (outpatient) decolonization therapy and the effectiveness of its single components could improve the acceptance of these measures.

### Limitations

The study was carried out in two regions in Germany and might not reflect the situation in all of Germany. While MRSA status was based on medical records, all further data on decolonization therapy and medical conditions were self-reported by MRSA carriers. Misclassification concerning application of control swabs, decolonization therapy or medical conditions e.g., due to recall problems cannot be excluded. Only 9.5 % of the physicians returned our questionnaire. Thus, our analysis is prone to selection bias, and the results have to be interpreted with caution. Knowledge, attitude, and activity might be higher among participants than among the whole source population. However, 22 % of responding physicians indicated that MRSA was not important in their daily work, and 10 % stated, that they had had no MRSA carriers among their patients in the last 12 months. Thus, we may assume that non-responders are not interested in taking part in studies in general and they are not specifically uninterested in MRSA. This may lead to less bias in our findings.

The response proportion of the MRSA carriers was 16.5 %. It can be assumed that in this population non-responders might be frailer than respondents, and thus face different problems concerning MRSA. Unfortunately, we are not able to show this. To approach these hard to reach populations further research with a different approach, e.g., interviews, would be necessary.

## Conclusion

Relevance attributed to MRSA and experience with application of MRSA specific therapy were highly heterogeneous among physicians in the outpatient sector. Raising the awareness towards MRSA in the outpatient healthcare sector appears imperative to improve treatment of MRSA carriers. Additionally, health care professionals should be aware of possible stigmatization and of the fact that lack of adequate information can result in inappropriate deprivation of social contacts. MRSA carriers need more and adequate information about MRSA. This information should include the fact that MRSA is no threat to healthy people.

### Ethics approval and consent to participate

The study was approved by the Ethics Committee of Hannover Medical School (No.1893-2013) and the Ethics Committee of the University Witten/Herdecke (No.112/2013). Participants provided informed consent by sending back the anonymous questionnaire.

### Availability of data and materials

The dataset supporting the conclusions of this article is available from the corresponding author upon request.

## Additional files

Additional file 1: Questionnaire for physicians working in the outpatient sector. (PDF 217 kb) Additional file 2: Questionnaire for MRSA carriers. (PDF 232 kb) Additional file 3: Figure S1. Recruitment of MRSA carriers. (PDF 178 kb)

## Abbreviations

CI: Confidence Interval
GP: general practitioner
IQR: interquartile range
MRSA: methicillin-resistant *Staphylococcus aureus*
OR: odds ratio
