# Supplementary material for: Care for MRSA carriers in the outpatient sector: a survey among MRSA carriers and physicians in two regions in Germany
Source: BMC Infect Dis. 2016 Apr 26;16:184. doi: 10.1186/s12879-016-1503-5 (PMC4845324; doi:10.1186/s12879-016-1503-5)
Supplement: Additional file 1: — Questionnaire for physicians working in the outpatient sector. (PDF 217 kb) [file 12879_2016_1503_MOESM1_ESM.pdf]

Additional file 1: Questionnaire for physicians working in the outpatient sector.

1	**How many MRSA positive patients have you seen in your practice in the last 12 months?** (If you don´t know the exact number, please estimate)<br>___________	Practice
2	**How relevant is MRSA with respect to your patients? Please give your answer on a scale from 0-10. “0” indicating “not relevant at all”, “10” indicating “very relevant”.**<br><br>0 1 2 3 4 5 6 7 8 9 10<br>☐ ☐ ☐ ☐ ☐ ☐ ☐ ☐ ☐ ☐ ☐<br>not all relevant very relevant	Attitude
3	**Which are your sources of information on MRSA?**<br>**Medical journals**<br>□Never □Rarely □Sometimes □Frequently □Very frequently<br>**Recommendations of the National Association of Statutory Health Insurance Physicians (“Kassenärztliche Bundesvereinigung”)**<br>□Never □Rarely □Sometimes □Frequently □Very frequently<br>**Recommendations of the Robert Koch Institute (RKI) (the central federal institution responsible for disease control and prevention)**<br>□Never □Rarely □Sometimes □Frequently □Very frequently<br>**Recommendations of the Medical Chamber (“Ärztekammer”)**<br>□Never □Rarely □Sometimes □Frequently □Very frequently<br>**Recommendations of a MRSA network**<br>□Never □Rarely □Sometimes □Frequently □Very frequently<br>**Conversation with colleagues**<br>□Never □Rarely □Sometimes □Frequently □Very frequently<br>**Other websites**<br>□Never □Rarely □Sometimes □Frequently □Very frequently	Information
4	**Provided you are familiar with the recommendations for MRSA in the outpatient sector of the RKI, the “Kassenärztliche Bundesvereinigung” or a MRSA network - do you agree with these recommendations**?<br>□Yes □In part □No □I don’t know these recommendations<br>□Don’t know	Recommendations
5	**Are you of the opinion that there is enough information available about MRSA in the outpatient sector?**<br>□Yes □No □ Don’t know	Information
6	**Are you a member of a MRSA quality circle or network?**<br>□Yes □No □Does not exist in my region	Practice
7	**Do you have a “MRSA certificate” from the National Association of Statutory Health Insurance Physicians?**<br>□Yes □No	Practice
8	**Do you know the reimbursement possibilities for the screening of patients at risk and the therapy of MRSA carriers?**<br>□Yes □No	Knowledge
9	**Which of the following criteria define a patient at risk according to the National Association of Statutory Health Insurance Physicians?** (Only two answers are correct)<br>□Hospitalization > 4 days in the last 6 months and MRSA positivity in case history	Knowledge

	□Hospitalization > 4 days in the last 6 months<br>□Living in a nursing home<br>□Hospitalization > 4 days in the last 6 months and the presence of at least two more risk factors (inserted catheter, need for care, chronic wounds, dialysis dependency, antibiotic therapy in the last 6 months)<br>□Antibiotic therapy in the last 4 weeks<br>□Don´t know	
10	**How many patients have you screened for MRSA in your practice in the last 12 months?** (If you don't know the exact number, please estimate**)**<br>___________	Practice
11	**A decolonization therapy has been thoroughly applied. From when on can you define your patient as permanently MRSA negative?** (Only one answer is correct)<br>□Negative swab 4 days to 4 weeks after completion of the decolonization therapy.<br>□Negative swab 3 months after completion of the decolonization therapy.<br>□Negative swabs at 2 time points: 3 to 6 months and 11 to 13 months after completion of the decolonization therapy.<br>□Negative swabs at 2 time points: 6 to 12 months and 22 to 24 months after completion of the decolonization therapy.	Practice
12	**How useful do you think is the application of a decolonization therapy in the outpatient sector in general?**<br>□Always useful □Often useful □Sometimes useful □Never useful	Attitude
13	**How many MRSA positive patients have you already treated with a decolonization therapy?**<br>(If you don't know the exact number, please estimate)<br>_________	Practice
14	**If you have already used decolonization therapies: How often do you estimate the decolonization therapy to have been successful?**<br>□Up to 0% □ Up to 33% □ Up to 66% □Almost 100% □Don´t know	Practice
15	**Are there, in your opinion, any difficulties concerning decolonization therapy in the outpatient sector?**<br>(Multiple answers possible)<br>□None<br>□Patients cannot procure the financial contribution necessary.<br>□Patients and family fail at the practical implementation.<br>□Staff at the nursing home fails at the practical implementation.<br>□Other:____<br>____	Practice
16	**How often do you estimate does the hospital actually report a positive MRSA finding to you?**<br>**In the discharge letter**<br>□Never □Rarely □Sometimes □Frequently □Very frequently<br>**Additionally by phone**<br>□Never □Rarely □Sometimes □Frequently □Very frequently	Information
17	**How often do you estimate to become informed about a decolonization therapy that was started at the hospital?**<br>□Never □Rarely □Sometimes □Frequently □Very frequently	Information
18	**Do you have specific requests concerning the communication between the inpatient and outpatient sector with regard to MRSA?**<br>____	Information

19	**Reimbursement for MRSA specific therapy was introduced on 31.03.2012; it allows reimbursement for:**<br>(multiple answers are possible)<br>□Determination of MRSA status for all patients > 60 years<br>□Therapy and care for a patient at risk who is MRSA positive or for a MRSA positive contact person<br>□MRSA status of a patient at risk<br>□Participation in a MRSA case conference or a regional MRSA network conference<br>□Remuneration of services is fixed, without upper limit and outside the patient's overall remuneration. ("außerhalb der morbiditätsbedingten Gesamtvergütung")	Knowledge
20	**I am entitled to the reimbursement of the above mentioned items.**<br>□Yes<br>□No<br>□Don't know	Reimbursement
21	**Costs for screening of patients at risk and their subsequent treatment can be claimed.**<br>**How satisfied are you with the amount of the reimbursement of the Statutory Health Insurance for the above mentioned items?**<br>□Very satisfied<br>□Satisfied<br>□Unsatisfied<br>□Don't know	Reimbursement
22	**Which adjustments do you recommend for reimbursement?**<br>____	Reimbursement
23	**You are a physician working in the outpatient sector. Which is your specialization?**<br>□General practitioner (GP)<br>□Internal medicine (working as GP)<br>□Internal medicine<br>□Dermatology<br>□Urologist<br>□Other	Demography
24	**Your gender is:**<br>□male □female	Demography
25	**Your age in years?**<br>__________	Demography
26	**For how many years have you been working as a physician in the outpatient sector?**<br>_________________	Demography
