# Supplementary material for: Care for MRSA carriers in the outpatient sector: a survey among MRSA carriers and physicians in two regions in Germany
Source: BMC Infect Dis. 2016 Apr 26;16:184. doi: 10.1186/s12879-016-1503-5 (PMC4845324; doi:10.1186/s12879-016-1503-5)
Supplement: Additional file 2: — Questionnaire for MRSA carriers. (PDF 232 kb) [file 12879_2016_1503_MOESM2_ESM.pdf]

Additional file 2: Questionnaire for MRSA carriers

1	**How many years has it been since you have been tested positive for MRSA for the first time?**<br>________ years ago □ Don't know	Medical History
2	**Have control swabs been taken outside the hospital?**<br>By the general practitioner? □Yes □No □Don't know<br>By another physician? □Yes □No □Don't know	Practice
3	**In which of the following places of the body has MRSA been found in your case?** Multiple answers are possible.<br>□On the skin<br>□In the nose<br>□In a wound<br>□Catheter or stomach tube or dialysis shunt<br>□In sputum (expectoration from airways)<br>□In blood or liquor (cerebrospinal fluid)<br>□Other:______________________<br>□Don't know	Medical History
4	**There are various contact partners within the health care system.**<br>**Who has talked to you about MRSA in the course of time?**<br>□General practitioner<br>□Other physician<br>□Medical doctor at a hospital<br>□Nursing staff at a hospital<br>□Staff of a home-care service<br>□Staff at a nursing home<br>□Other:____________________________________<br>□None	Information
5	**How did you obtain information about MRSA outside the medical sector?**<br>Multiple answers are possible.<br>□Family / friends<br>□Newspaper<br>□TV/radio<br>□Internet<br>□Other	Information
6	**How well do you feel informed about MRSA?**<br>**Please give your answer on the scale from 0-10.**<br>**0 indicating "I don't feel informed at all"**<br>**10 indicating "I feel very well informed"**<br><br>0 1 2 3 4 5 6 7 8 9 10<br>□ □ □ □ □ □ □ □ □ □ □<br>Not informed at all Very well informed	Information
7	**MRSA colonization means:**<br>**This bacterium is on my skin or mucosa and is not dangerous at the moment.**<br>□Correct □Not correct □Don't know	Knowledge
8	**MRSA colonization means: I am sick**<br>□Correct □Not correct □Don't know	Knowledge
9	**In case of healthy individuals, MRSA often disappears automatically over time.**<br>□Correct □Not correct □Don't know	Knowledge
10	**There are no antibiotics at all to treat an illness caused by MRSA.**	Knowledge

	□Correct □Not correct □Don't know	
11	**Contact to healthy individuals in the private setting is no problem. This is also true for skin contact, i.e. handshaking or hugging.**<br>□Correct □Not correct □Don't know	Knowledge
12	**When dealing with infected wounds, special care should be applied (i.e. the use of gloves and hand disinfections)**<br>□Correct □Not correct □Don't know	Knowledge
13	**If MRSA was diagnosed, which of the following home hygiene measures is recommended? (only one correct answer)**<br>□Laundry should be washed at ≥90°C.<br>□Laundry washed at 60°C and the use of all-purpose detergent ("Vollwaschmittel") is sufficient.<br>□Laundry washed at 30°C and the use of all-purpose detergent is sufficient.	Knowledge
14	**How would you describe your current state of health?**<br>□Excellent □Very good □Good □Not so good □Bad	State of health
15	**How satisfied are you with your current quality of life in general?**<br>□Very satisfied □Satisfied □Rather satisfied □Rather unsatisfied □Unsatisfied	Life quality
16	**Has your quality of life change because of MRSA?**<br>□Improved □Rather improved □Unchanged □Rather worsened □Worsened	Life quality
17	**Do you attach importance to the MRSA finding?**<br>□Yes, a lot □Yes, some □Intermediate □Rather not □No	Attitude
18	**Does the MRSA finding make you afraid?**<br>□Yes, a lot □Yes, some □Intermediate □Rather not □No	Attitude
19	**Does MRSA influence your daily life at home (not at the hospital)?**<br>**I have given up activities because of MRSA (i.e. swimming or other sport, sauna)**<br>□Does apply □Does not apply	Stigmatization
	**I was rejected by an ambulance service because of MRSA.**<br>□Does apply □Does not apply	Stigmatization
	**I was rejected by a dentist or resident physician because of MRSA.**<br>□Does apply □Does not apply	Stigmatization
	**I was rejected by a nursing home because of MRSA.**<br>□Does apply □Does not apply	Stigmatization
	**I was rejected by a rehabilitation clinic because of MRSA.**<br>□Does apply □Does not apply	Stigmatization
	**I avoid other people due to MRSA because I don't want to transmit the germ [i.e. grandchildren].**<br>□Does apply □Does not apply	Stigmatization
	**I feel avoided by others (e.g. neighbours) because of MRSA.**<br>□Does apply □Does not apply	Stigmatization
20	**Which of the following procedures was applied to you during the decolonization therapy?**<br>**Therapy with nasal ointment**<br>□Yes □No □Don't know	Practice
	**Skin and Hair washing with disinfectants**<br>□Yes □No □Don't know	Practice
	**Mouthwashes**<br>□Yes □No □Don't know	Practice
	**Daily change of bedding and towels**<br>□Yes □No □Don't know	Practice

	**Daily change of clothing**<br>□Yes □No □Don't know	Practice
	**Daily change of hygiene utensils like toothbrush, razor, comb etc.**<br>□Yes □No □Don't know	Practice
21	**Has the application of this special therapy to remove MRSA been proposed to you?**<br>In the hospital □Yes □No □Don't know<br>By a physician after discharge from hospital □Yes □No □Don't know	Practice
22	**Has this special therapy been applied to you?**<br>□Yes □No Why not?______________________________	Practice
	**If yes, where?**<br>In the Hospital □Yes □No □Don't know<br>At home □Yes □No □Don't know<br>Started in the hospital and terminated at home □Yes □No □Don't know	Practice
23	**This special therapy usually takes 5 days.**<br>**Has this therapy been applied to you more than once?**<br>□Yes ----times<br>□No<br>□Don't know	Practice
24	**Has this special therapy been successful?**<br>□Yes □No □Don't know	Practice
25	**Do you remember the result of the last swab?**<br>Was it:<br>□Positive for MRSA □Negative for MRSA □Don't know	Medical History
26	**Did you encounter any problems during this therapy? Which ones?**<br>____	Practice
27	**Patients have to bear a proportion of the costs by themselves, i.e. for the special shampoo and washing lotion. Which answer would apply to you?**<br>□The additional costs **are a problem** for me.<br>□The additional costs **are no problem** for me.<br>□Don't know.	Practice
28	**Would you like to tell us something else which we forgot?**<br>____	
29	**Your gender is:**<br>□Male □Female	Demography
30	**How old are you in years?**<br>___________ years	Demography
31	**Do you live in a nursing home?**<br>□Yes □No	Demography
32	**Do you have a constant need for nursing care ("Pflegestufe")**<br>**□Yes □No**	Demography
33	**Does one or more of the following risk factors apply to you?**<br>(Multiple answers are possible)<br>□Urinary catheter<br>□Dialysis<br>□Chronic open wounds<br>□A chronic skin disease, e.g. psoriasis<br>□Occupational exposure to lifestock, e.g. as a farmer or veterinarian	Medical history
34	**Which is your highest school leaving qualification?**<br>Primary education<br>Lower secondary general education, without direct access to upper secondary education ("Hauptschule")	Demography

	Lower secondary general education, with direct access to upper secondary education ("Realschule")<br>Lower secondary general education, with direct access to upper secondary education ("Polytechnische Oberschule" in the German Democratic Republic)<br>Upper secondary general education, with direct access to universities of applied sciences ("Fachhochschulreife")<br>Upper secondary general education, with direct access to universities ("allgemeine Hochschulreife")<br>Other school leaving qualification:<br>Still in school, aspired school leaving qualification:	
35	**In which country were you born?**<br>□In Germany (within the current national borders)<br>□In Germany (within the borders before 1945)<br>□In another country	Demography
36	**Is German your mother tongue?**<br>□Yes □No	Demography
36a	**If German is not your mother tongue, how good is your command of the German language?**<br>□very good □good □moderate □little □none	Demography
37	**Did you need help to complete the questionnaire?**<br>□Yes □No	Demography
