# Supplementary figures and images for: Care for MRSA carriers in the outpatient sector: a survey among MRSA carriers and physicians in two regions in Germany
Source: BMC Infect Dis. 2016 Apr 26;16:184. doi: 10.1186/s12879-016-1503-5 (PMC4845324; doi:10.1186/s12879-016-1503-5)

**Additional Figure 1: Recruitment of MRSA carriers**

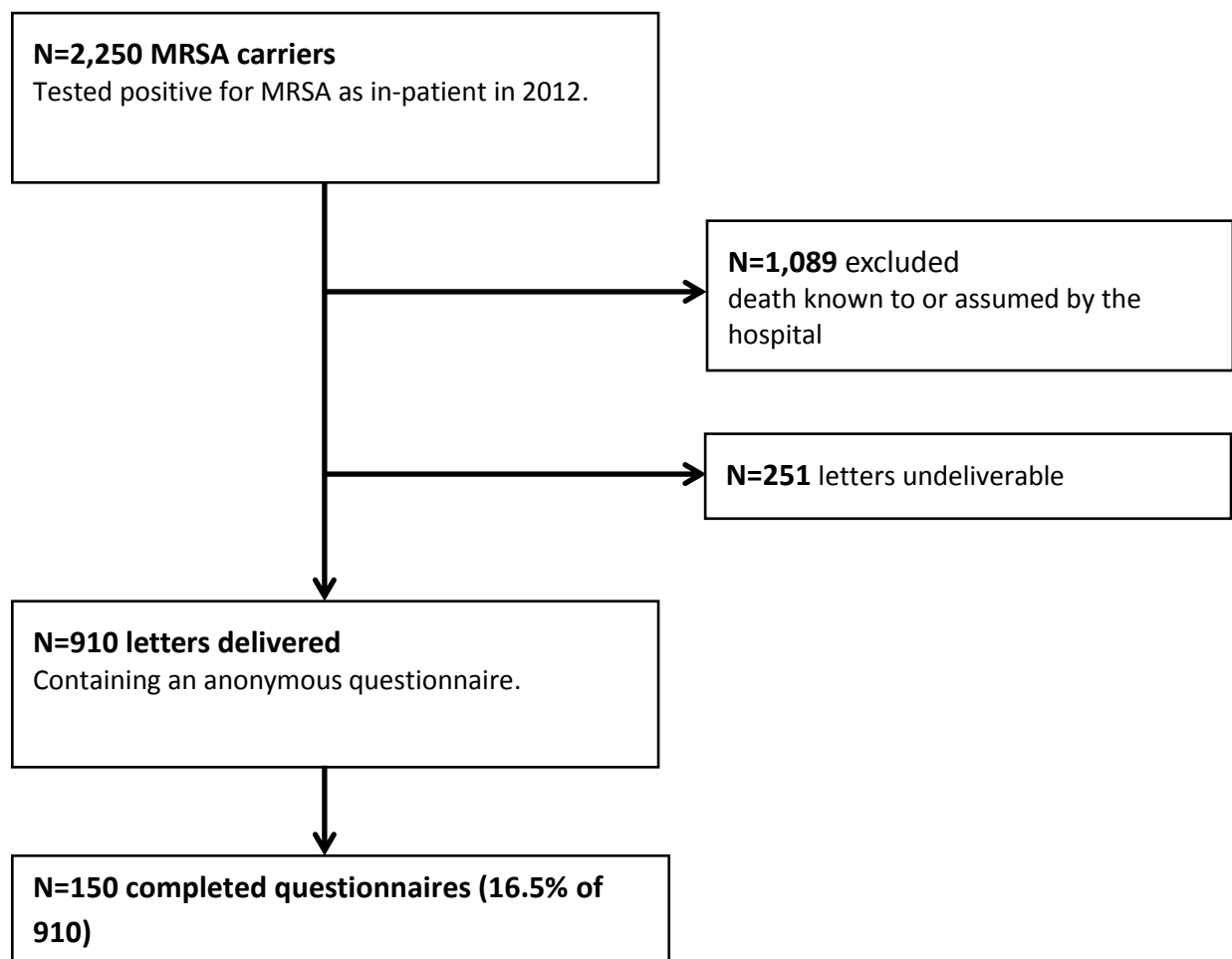

Supplement: Additional file 3: Figure S1. — Recruitment of MRSA carriers. (PDF 178 kb) [file 12879_2016_1503_MOESM3_ESM.pdf]
